# Iron Administration Overcomes Resistance to Erastin-Mediated Ferroptosis in Ovarian Cancer Cells

**DOI:** 10.3389/fonc.2022.868351

**Published:** 2022-03-31

**Authors:** Anna Martina Battaglia, Alessandro Sacco, Ida Daniela Perrotta, Maria Concetta Faniello, Mariangela Scalise, Daniele Torella, Sonia Levi, Francesco Costanzo, Flavia Biamonte

**Affiliations:** ^1^Laboratory of Biochemistry and Cellular Biology, “Magna Graecia” University of Catanzaro, Department of Experimental and Clinical Medicine, Catanzaro, Italy; ^2^Laboratory of Transmission Electron Microscopy, University of Calabria, Department of Biology, Ecology and Earth Sciences, Centre for Microscopy and Microanalysis, Cosenza, Italy; ^3^Laboratory of Molecular and Cellular Cardiology, “Magna Graecia” University of Catanzaro, Department of Experimental and Clinical Medicine, Catanzaro, Italy; ^4^School of Medicine, Vita-Salute San Raffaele University, Milan, Italy; ^5^Division of Neuroscience, Istituto di Ricovero e Cura a Carattere Scientifico (IRCCS) San Raffaele Scientific Institute, Milan, Italy; ^6^Magna Graecia University of Catanzaro, Interdepartmental Centre of Services, Catanzaro, Italy

**Keywords:** ferroptosis, ovarian cancer, iron, erastin, chemoresistance, mitochondrial dysfunction

## Abstract

**Objectives:**

Developing novel therapeutic approaches to defeat chemoresistance is the major goal of ovarian cancer research. Induction of ferroptosis has shown promising antitumor effects in ovarian cancer cells, but the existence of still undefined genetic and metabolic determinants of susceptibility has so far limited the application of ferroptosis inducers *in vivo*.

**Methods:**

Erastin and/or the iron compound ferlixit were used to trigger ferroptosis in HEY, COV318, PEO4, and A2780CP ovarian cancer cell lines. Cell viability and cell death were measured by MTT and PI flow cytometry assay, respectively. The “ballooning” phenotype was tested as ferroptosis specific morphological feature. Mitochondrial dysfunction was evaluated based on ultrastructural changes, mitochondrial ROS, and mitochondrial membrane polarization. Lipid peroxidation was tested through both C11-BODIPY and malondialdehyde assays. VDAC2 and GPX4 protein levels were quantified as additional putative indicators of mitochondrial dysfunction or lipid peroxidation, respectively. The effect of erastin/ferlixit treatments on iron metabolism was analyzed by measuring intracellular labile iron pool and ROS. FtH and NCOA4 were measured as biomarkers of ferritinophagy.

**Results:**

Here, we provide evidence that erastin is unable to induce ferroptosis in a series of ovarian cancer cell lines. In HEY cells, provided with a high intracellular labile iron pool, erastin treatment is accompanied by NCOA4-mediated ferritinophagy and mitochondrial dysfunction, thus triggering ferroptosis. In agreement, iron chelation counteracts erastin-induced ferroptosis in these cells. COV318 cells, with low baseline intracellular labile iron pool, appear resistant to erastin treatment. Notably, the use of ferlixit sensitizes COV318 cells to erastin through a NCOA4-independent intracellular iron accumulation and mitochondrial dysfunction. Ferlixit alone mimics erastin effects and promotes ferroptosis in HEY cells.

**Conclusion:**

This study proposes both the baseline and the induced intracellular free iron level as a significant determinant of ferroptosis sensitivity and discusses the potential use of ferlixit in combination with erastin to overcome ferroptosis chemoresistance in ovarian cancer.

## Introduction

Ovarian cancer (OVCA) is the hardest-to-treat gynecologic malignancy worldwide (1). During the last decade, many novel anticancer agents, such as molecularly targeted drugs (i.e., bevacizumab or PARP-inhibitors) and combination therapies have been clinically validated; however, the overall survival rate has not been improved, since a large number of ovarian tumors, afterwards initial responsiveness, become chemo-resistant (2, 3). Ferroptosis, a new non-apoptotic form of regulated cell death featured by lipid peroxidation and/or mitochondrial dysfunction, has been recently proposed as a potential therapeutic opportunity for many therapy-resistant cancer cells (4). Indeed, preclinical studies have demonstrated that ferroptosis inducers (FINs) may enhance the chemosensitivity of OVCA (5). To make a few examples, erastin may increase the OVCA cells sensitivity to docetaxel by reducing the drug-efflux activity of ABCB1 (6). Artesunate (ART) promotes a strong induction of reactive oxygen species (ROS) thus inhibiting the *in vitro* proliferation of ovarian cancer cells and the tumor growth in the relative mouse models (7). The PARP-inhibitor olaparib represses SLC7A11 and thus synergizes with erastin or sulfasalazine to boost ferroptosis in HEY and A2780 cells (8). However, FINs have so far shown the limit of being effective only in a subset of OVCA (9). This phenomenon has been associated with the high intra-tumor heterogeneity of this disease, based on different somatic mutations, nutrient preferences, and environmental stimuli (5). Hence, the identification of genetic, metabolic, or environmental factors enabling the selection of the best-responders to ferroptosis-inducing therapies remains a critical step to improve treatment in ovarian cancer.

*KRAS* mutation is the first genetic determinant found to sensitize cancer cells to erastin-induced ferroptosis (4). *KRAS*-mutated cells may show high levels of the transferrin receptor 1 (TFR1) and decreased expression of the ferritin heavy chain (FtH), thus leading to enhanced iron intake, inhibited iron storage, and accumulation of the labile iron pool (LIP) (10). As expected, the intracellular free Fe^2+^ triggers Fenton reactions and promotes the generation of ROS, thus making cancer cells more vulnerable to ferroptosis (11). Similarly, enhanced NCOA4-mediated autophagic degradation of ferritin (i.e., ferritinophagy) results in LIP and ROS accumulation, ultimately promoting ferroptotic cell death (12). Conversely, the activation of the NRF2 pathway or the inhibition of the serine/threonine kinase ATM represses ferroptosis by promoting iron storage through the transcriptional activation of *FtH* (13, 14). Recently, Torti’s group proposed that OVCA cells showing a so-called “iron-addicted” phenotype, characterized by a marked iron uptake and a weak iron efflux, are more susceptible to erastin both *in vitro* and *in vivo* (15). However, further evidence is necessary to confirm that iron addiction may represent a metabolic determinant of ferroptosis sensitivity that can be therapeutically exploited in OVCA.

Another unsolved issue regarding the rational application in clinical practice of FINs is the existence of several chemical and physical weaknesses such as poor pharmacokinetic, low water solubility and unstable metabolism (16, 17). To make an example, sulfasalazine (SAS), an FDA approved class I FIN able to inhibit SLC7A11, induces ferroptosis in breast and head and neck cancer (18, 19), but its clinical use in OVCA remains poor (20). The development of new combinational strategies that can synergistically work with FINs represents one of the potential solutions to this issue (17). In this regard, both docetaxel and PARP inhibitors have been proved to potentiate erastin- or RSL3-induced cell death in OVCA cells (21).

Here, we show that erastin efficacy in OVCA cells might be independent of *KRAS* mutational status and of the responsiveness to conventional chemotherapeutic drugs (i.e., cisplatin). Rather, we demonstrate that sufficient constitutive and treatment-induced amount of LIP is mandatory for erastin-mediated ferroptosis and that the use of ferlixit, an iron compound normally used to treat patients suffering anemia, overcomes erastin-mediated ferroptosis resistance. Overall, in this study, we validate the concept of “iron addiction” as a determinant of ferroptosis susceptibility in OVCA cells and we propose the combined use of ferlixit and erastin as a new therapeutic approach.

## Materials and Methods

### Cell Lines and Cell Culture

The human epithelial ovarian cancer cell lines HEY and COV318 were purchased from the American Type Culture Collection (ATCC, Rockville, MD, USA). COV318 and HEY cells were grown in DMEM medium (Sigma-Aldrich, St. Louis, MO, USA) supplemented with 10% (v/v) fetal bovine serum (FBS) (Invitrogen, San Diego, CA), L-glutamine (0.3 g/l) and 1% (v/v) penicillin and streptomycin (Sigma-Aldrich, St. Louis, MO, USA) at 37°C in a humidified incubator with 5% CO_2_ atmosphere. The cisplatin-resistant ovarian cancer cell lines (PEO4 and A2780CP) were obtained from the American Type Culture Collection (ATCC, Rockville, MD, USA). These cells were cultured in RPMI 1640 (Sigma-Aldrich, St. Louis, MO, USA), supplemented with 10% (v/v) fetal bovine serum (FBS) (Invitrogen, San Diego, CA), L-glutamine and 1% (v/v) penicillin and streptomycin (Sigma-Aldrich, St. Louis, MO, USA) in adherent cultures at 37°C in a humidified incubator with 5% CO_2_ atmosphere. To maintain the resistance, 1 µM cisplatin was added to the medium of A2780CP and PEO4 cells every two to three passages. All cell lines were tested for mycoplasma contaminations and STR profiled for authentication.

### Reagents

Ferlixit (62.5 mg/5 ml, sodium ferric gluconate complex in sucrose, SANOFI) and cisplatin (50 mg/100 ml, SANDOZ) were obtained from the outpatient pharmacy at the Unit of Gynecologic Oncology, Magna Graecia University of Catanzaro; deferoxamine mesylate salt (DFO), erastin, ferrostatin-1 (Fer-1), hemin and cloroquine (Cq) were purchased from Sigma Aldrich (Sigma-Aldrich, St. Louis, MO, USA); the antioxidant (±)-6-hydroxy-2,5,7,8-tetra-methylchromane-2-carboxylic acid (trolox) was ordered from Cayman Chemical (Cayman Chemical Company, Ann Arbor, USA). Cells were seeded in a 6-well plate in serum-free medium. Each compound was used at the following final concentrations: ferlixit at 100 µM and 250 µM for 1, 8, 24 or 48 h; cisplatin at 150 μM for 24 h; DFO at 200 µM for 24 h; erastin at 8 µM and 25 µM for 1, 8 or 24 h; Fer-1, hemin and Cq at 10 µM for 8, 24 or 48 h; and trolox at 200 μM for 8 h. Treatments were performed at least three times on independent biological replicates.

### Cell Viability Assay

3-[4,5-Dimethyl-2-thiazolyl]-2,5-diphenyltetrazolium bromide (MTT) (Sigma Aldrich, St. Louis, MO, USA) assay was performed to detect proliferation of HEY and COV318 cells untreated or treated with erastin, cisplatin and ferlixit. A total of 10^4^ cells/well were seeded into a 96-well flat-bottom plate and were subjected to drug treatment. There were quintuplicates for each cell type. Fresh MTT (5  mg/ml, Sigma Aldrich, St. Louis, MO, USA), re-suspended in phosphate-buffered saline (PBS) was added to each well for a final concentration of 0.5 mg/ml. After 4 h incubation, culture medium was discarded and replaced with 200 μl of isopropanol to solubilize formazan crystals. Optical density was measured at 595 nm in a spectrophotometer. Each experiment was performed in triplicate.

### Cell Counting

Growth rate of HEY and COV318 cells was obtained using the trypan blue dye exclusion method. Cells were counted after 24, 48, and 7h. Each experiment was performed in triplicate.

### PI Staining Analysis

Approximately 1 × 10^6^ cells/well were seeded in 6-well plates overnight followed by the various treatment. Cells were centrifuged and the relative pellets were incubated with PI staining in the dark at 37°C for 15 min. Samples were then washed twice with PBS. The cellular fluorescence was analyzed by FACS BD LSRFortessaTM X-20 cytofluorometer (BD Biosciences). A total of 2 × 10^4^ event was acquired for each sample from three independent experiments. Fluorescence was measured using FlowJo software program (Tree Star, Inc.). Each experiment was performed in triplicate.

### Optical Microscopy of “Ballooning” Phenotype

Cells were seeded in 6-well plates at density of 4 × 10^5^ cells/well and grown overnight. Following treatment with erastin, ferlixit, hemin, and DFO, morphological changes of “ballooning” phenotype were observed by light microscopy. Images were captured with Leica DM IL LED inverted phase contrast microscope (Leica Microsystems, Wetzlar, Germany).

### Transmission Electron Microscopy (TEM) Measurements of Cellular Ultrastructural Morphological Changes

HEY and COV318 cells (2 × 10^6^ cells/well) were plated in 100 mm culture dishes. Cells were then treated with erastin, DFO and/or ferlixit. Cells were centrifuged and the relative pellet were fixed for 3 h with 3% glutaraldehyde solution in 0.1 M phosphate buffer (pH 7.4). After washing in PBS for 15 min, samples were post-fixed in osmium tetroxide (1%) for 2 h, dehydrated in graded acetone, and then progressively embedded in acetone/resin with final embedment in pure resin (Araldite‐Fluka). Ultrathin sections (60–90 nm in thickness) were cut with a diamond knife, collected on copper grids (G300 Cu), and then examined with a Jeol JEM 1400‐Plus electron microscope operating at 80 kV.

### Measurement of Intracellular ROS

Intracellular ROS amounts were determined by incubating cells for 10 min at 37°C with the redox-sensitive probe 2′-7′-Dichlorodihydrofluorescein diacetate (CM-H2DCFDA; Thermo Fisher Scientific, Waltham, USA), according to the instructions of the manufacturer. CM-H2DCFDA fluorescence was analyzed by flow cytometry using a FACS BD LSRFortessaTM X-20 cytofluorometer (BD Biosciences) and data were processed with FlowJo software (Tree Star, Inc.). Each experiment was performed in triplicate.

### BODIPY™ Assay

Lipid peroxidation was investigated by flow cytometry using BODIPY™ 581/591 C11 dye (Thermo Fisher Scientific, Waltham, USA). Briefly, cells were seeded in 6-well plates at a density of 4 × 10^5^ cells/well and grown overnight. After treatments, cells were loaded with 2.5 μM BODIPY™ 581/591 C11 for 30 min at 37°C. After 30 min of loading, unincorporated dye was removed by washings twice with PBS. Samples were then centrifuged at 1,000 r.p.m. for 3 min and the pellets were resuspended in 500 μl of PBS. The cell suspension was subjected to the flow cytometry analysis to analyze the amount of lipid ROS within cells. Oxidation of BODIPY-C11 resulted in a shift of the fluorescence emission peak from ~590 to ~510 nm proportional to lipid ROS generation (22). The fluorescence intensities of cells per sample were determined by flow cytometry using the FACS BD LSRFortessaTM X-20 cytofluorometer (BD Biosciences). A minimum of 20,000 cells were analyzed per condition. Fluorescence of each probe was measured using FlowJo software program (Tree Star, Inc.). Each experiment was performed in triplicate.

### Lipid Peroxidation Assay (Malondialdehyde, MDA)

Cellular MDA levels were determined by using the lipid peroxidation assay kit (Sigma Aldrich, MAK085, Missouri, USA) according to the protocol of the manufacturer. Briefly, cells were seeded in a 6-well plate (5 × 10^5^ cells per plate) and treated with erastin or ferlixit. Cells were washed with ice-cold PBS and homogenized on ice in 300 μl of the MDA lysis buffer with 3 μl BHT (100×), then centrifuged (13,000×*g*, 10 min) to remove insoluble material. Approximately 200 μl of the supernatant from each homogenized sample were placed into a microcentrifuge tube; then 600 μl of the thiobarbituric acid (TBA) solution were added into each vial to form the MDA-TBA adduct. The mixture was incubated for 1 h at 95°C followed by ice-cooling for 10 min. Lipid peroxidation was determined by the reaction of MDA with thiobarbituric acid (TBA) to form a colorimetric (532 nm)/fluorometric adduct, proportional to the MDA present. The absorbance was measured in a spectrophotometer. Each experiment was performed in triplicate.

### Mitochondrial ROS Analysis

Generation of mitochondrial ROS was measured by flow cytometry with the use of MitoSOX Red Mitochondrial Superoxide Indicator (Thermo Fisher Scientific Inc.). After treatments, cells were incubated with 5 µM MitoSOX Red for 10 min at 37°C and then analyzed by flow cytometry using a FACS BD LSRFortessaTM X-20 cytofluorometer (BD Biosciences). A minimum of 20,000 cells was analyzed per condition. Fluorescence was measured using FlowJo software program (Tree Star, Inc.). Each experiment was performed in triplicate.

### Measurement of Mitochondrial Membrane Potential

Changes in the mitochondrial membrane potential were analyzed by staining the cells with TMRE (tetramethylrhodamine ethyl ester) dye (Thermo Fisher Scientific, Waltham, USA). Briefly, cells were cultured in 6-well plates and, upon treatments, were incubated with 100 nM TMRE dye for 30 min at 37°C and then washed with PBS. Samples were then centrifuged at 1,000 r.p.m. for 3 min and the pellets were resuspended in 500 μl of PBS. TMRE fluorescence was analyzed by flow cytometry using a FACS BD LSRFortessaTM X-20 cytofluorometer (BD Biosciences). A minimum of 20,000 cells was analyzed per condition. Fluorescence was measured using FlowJo software program (Tree Star, Inc.). Each experiment was performed in triplicate.

### Measurement of the LIP Level

The intracellular labile iron pool (LIP) was quantified by monitoring the recovering of calcein fluorescence induced by iron chelators after the calcein fluorescence was quenched by intracellular LIP. Briefly, cancer cells were seeded in 6-well plates at a density of 4 × 10^5^ cells/well and grown overnight. Then cells were loaded with 0.25 μM calcein acetoxymethyl ester (CA-AM; calcein-AM) (Sigma-Aldrich, Missouri, USA) for 30 min at 37°C. After washing twice with PBS, cells were treated with 200 μM 3-hydroxy-1,2-dimethyl-4(1H)-pyridone (deferiprone or L1) (Sigma-Aldrich, Missouri, USA) or left untreated. Following staining, and washing PBS, cells were analyzed by a FACS BD LSRFortessaTM X-20 cytofluorometer (BD Biosciences). The difference in the mean fluorescence index between chelator-treated and untreated cells (Δ mean fluorescence intensity, ΔMFI) reflects the amount of LIP. Each experiment was performed in triplicate.

### Total, Nuclear, and Cytoplasmic Protein Extraction

To obtain total protein extracts cells were washed once with PBS (1×) and total cell lysates were prepared using RIPA buffer containing 1 M Tris HCl, Triton X-100, 3 M NaCl, 0.5 M EDTA, 10% SDS supplemented with cOmplete™ Protease Inhibitor Cocktail provided in EASYpacks (Roche Diagnostics, Mannheim, Germany) as described by Biamonte et al. (23). Briefly, extracts were centrifuged at 12,000×*g* for 30 min at 4°C to eliminate insoluble fragments. For nuclear and cytoplasmic protein extraction, 2 × 10^7^ cells were washed once with PBS (1×) and cell pellets were resuspended in 200 μl of Lysis Buffer w/NP40. After 5 min incubation in ice, all tubes were centrifuged at 3,500 r.p.m. for 5 min at 4°C. The supernatant, containing cytoplasmic protein fraction, was transferred in a new eppendorf tube, while the pellet, containing cell nuclei, was washed in PBS (1×). To obtain nuclear protein content, the latter was resuspended in 1 ml of Lysis Buffer w/o NP40 and centrifuged twice at 3,500 r.p.m. at 4°C for 5 and 10 min, respectively. After these two centrifugations, the pellet was resuspended in 300 μl of Lysis Buffer w/o NP40 and 300 μl of Cushing Buffer were added. This mix solution was centrifuged at 6,000 r.p.m. for 10 min at 4°C and then resuspended in 300 μl of Resuspension Buffer. Three freeze-thaw cycles were performed according to protocol. Following a last centrifuge at 9,500 r.p.m. for 15 min at 4°C, the supernatant, containing nuclear proteins was collected. For recipes of buffers used for selective nuclear and cytoplasmic protein extraction refer to Chirillo et al. (24).

### Western Blotting

Protein amount was determined using the Protein Assay Dye Reagent Concentrate (Bio-Rad Laboratories, Hercules, California, USA). Each protein sample (40–50 μg) was separated by 10–15% SDS–PAGE and then transferred to nitrocellulose membranes (Sigma-Aldrich, St. Louis, MO, USA). After blocking with 5% milk, incubation with primary antibody was performed overnight at 4°C. Antibodies against FtH (1:200, sc-376594), NCOA4 (1:500, sc-373739) were purchased from Santa Cruz Biotechnology (Santa Cruz Biotechnology, Dallas, Texas); antibodies against VDAC2 (1:500, ab37985) and GPX4 (1:500, ab41787) were purchased from Abcam (Cambridge, United Kingdom). After incubation with peroxidase-conjugated secondary antibodies (Peroxidase AffiniPure Sheep Anti-Mouse IgG, 1:10,000; Peroxidase AffiniPure Donkey Anti-Rabbit IgG, 1:10,000; Peroxidase AffiniPure Donkey Anti-Goat IgG, 1:10,000; Jackson ImmunoResearch Europe Ltd. Cambridge House) for 1 h at room temperature, the signals were visualized *via* using the ECL western blotting detection system (Santa Cruz Biotechnology, Dallas, Texas) and acquired by Uvitec Alliance Mini HD9 (Uvitec Cambridge, UK). To ensure equal loading of proteins a goat polyclonal anti-γ-Tubulin (γ-TUB) antibody (1:3,000; sc-17787; Santa Cruz Biotechnology) was used. The protein band intensity on western blots was quantified and normalized to that of γ-TUB by using ImageJ software (http://rsb.info.nih.gov/ij/).

### Quantitative Real-Time Reverse Transcription (qRT)-PCR

Total RNA was extracted using the Trizol method (Life Technologies, Carlsbad, CA, USA) according to the instructions of the manufacturer (25, 26). Then, 1 µg of total RNA were retrotranscribed using High-Capacity cDNA Reverse Transcription Kit (Thermo Fisher Scientific, Waltham, Massachusetts, USA). qRT-PCR was performed using the SYBR Green qPCR Master Mix (Thermo Fisher Scientific, Waltham, Massachusetts, USA). Analysis was performed on QuantStudio 3 Applied Biosystems by Thermo Fisher Scientific. The relative mRNA expression level of *p53* and *BAX* were calculated by the 2^−ΔΔCt^ method and glyceraldehyde 3-phosphate dehydrogenase (*GAPDH*) was used as the housekeeping gene. Each experiment was performed in triplicate.

### Statistical Analysis

Overall data are represented as mean ± standard deviation (SD) of at least three biological replicates. When appropriate, data were analyzed by performing a simple comparison between two groups using Student’s *t-*test. We were interested in determining whether the means of more than two groups were equal or not, thus, we performed an analysis of variance (ANOVA). A *p*-value <0.05 was considered statistically significant.

## Results

### Erastin is not a *Bona Fide* Ferroptosis Inducer in Ovarian Cancer Cell Lines

**Figure 1:**
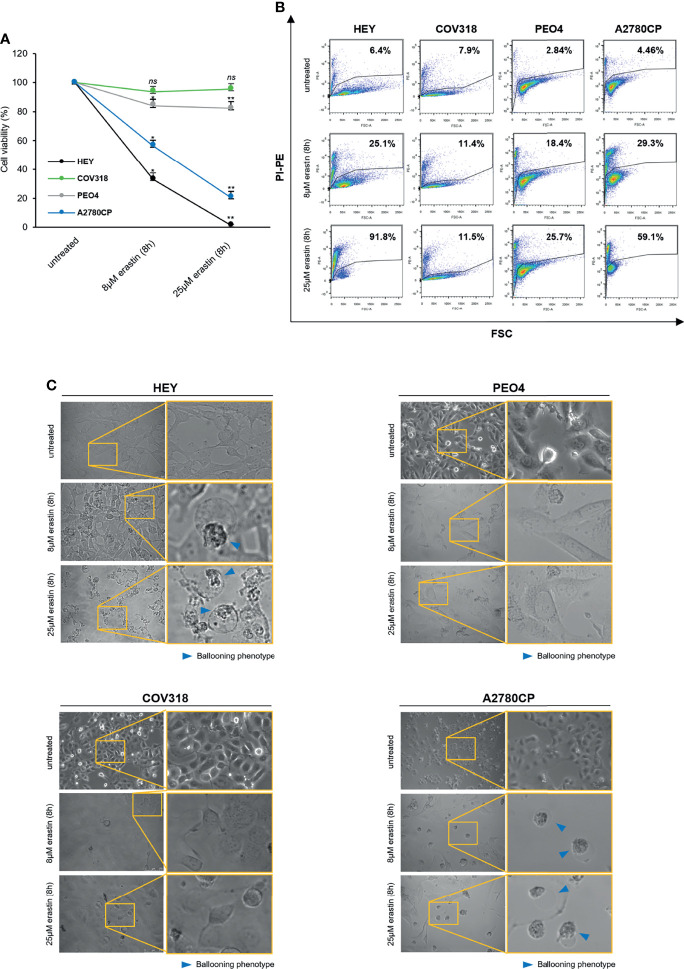
Erastin induces ferroptosis only in a subset of OVCA cells. HEY, COV318, PEO4, and A2780CP OVCA cells were treated with different concentrations (8 and 25 µM) of erastin for 8 h. Cell viability and mortality were measured using the MTT assay **(A)** and the PI flow cytometric analysis **(B)**, respectively. Results are shown as means ± SD of three independent experiments. *p-value <0.05, untreated vs 8 µM erastin (8 h); **p-value <0.05, untreated vs 25 µM erastin (8 h); ns: not significant. **(C)** Optical microscopy images showing the presence or absence of the ballooning phenotype upon treatment with erastin (8 and 25 µM for 8 h).

Ferroptosis has been proposed as a new potential therapeutic option for both chemotherapy-sensitive and -resistant OVCA (5, 21), but its effectiveness is still currently limited, also due to a lack of insights on the genetic and metabolic determinants of ferroptosis sensitivity. Here, we first assessed whether erastin is a *bona fide* ferroptosis inducer in OVCA cell lines. Taking into account that erastin was originally selected as an agent able to trigger ferroptosis exclusively in *RAS*-mutated cancer cells, we selected four OVCA cell lines, three of which are *KRAS*^wt^ (COV318, PEO4, and A2780CP) and one is *KRAS*^G12D^ (HEY). Furthermore, this panel includes both cisplatin-sensitive (HEY and COV318) and cisplatin-resistant (PEO4 and A2780CP) cells (see cell viability assay in **Supplementary Figure S1**). HEY, COV318, PEO4, and A2780CP cells were treated with two increasing concentrations of erastin (8 and 25 μM) for 8 h. As shown in **Figure 1A**, HEY and A2780CP cell viability significantly decreased upon treatment with 8 μM (~35–55%, *p <*0.05) and further broke down with 25 μM erastin for 8 h (~1–20%, *p <*0.05). Conversely, COV318 and PEO4 cells are insensitive to both erastin concentrations, with cell viability nearly of 90% upon treatments. Similar results were obtained by using propidium iodide (PI) staining. Flow cytometry analysis highlighted a significant increase in cell death in HEY and A2780CP cells treated with both 8 μM (25.1% PI^+^ HEY and 29.3% PI^+^ A2780CP cells) and 25 μM erastin (91.8% PI^+^ HEY and 59.1% PI^+^ A2780CP cells) for 8 h (**Figure 1B**) while no significant differences in cell death were observed in COV318 and PEO4 cells upon treatment. In agreement, morphological observation under inverted phase-contrast microscope identified in treated-HEY and A2780 cells the ferroptosis distinct “ballooning” phenotype (**Figure 1C**). This morphological feature was undetectable in COV318 and PEO4 cells. These results indicate that erastin is not always effective in OVCA cells and that the erastin-sensitivity is after all independent of either *KRAS* mutational status or cisplatin sensitivity.

### Erastin Fails to Trigger Ferroptosis in Ovarian Cancer Cells With Low Intracellular LIP

**Figure 2:**
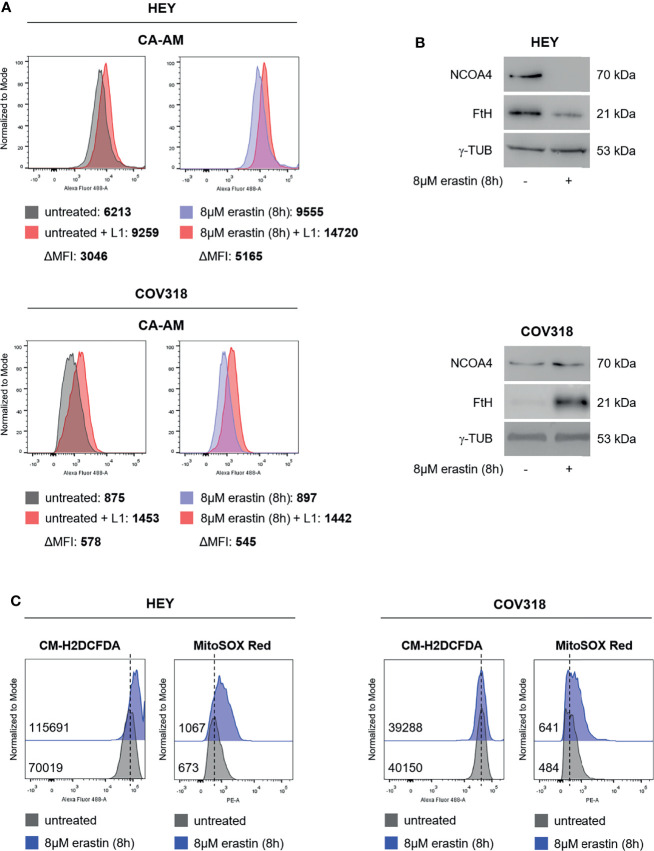
Erastin promotes ferroptosis in HEY cells, but not in COV318 cells. **(A)** LIP level assessed by flow cytometry using CA-AM in HEY and COV318 cells upon treatment with 8 µM erastin for 8 h. **(B)** Western Blot analysis of NCOA4 and FtH in HEY and COV318 cells untreated or treated with 8 µM erastin for 8 h. γ-TUB was used as internal control. **(C)** Flow cytometry analysis of cytosolic and mitochondrial ROS production after erastin administration (8 µM erastin for 8 h). Cytosolic ROS were measured upon staining with CM-H2DCFDA while mitochondrial ROS were quantified by using MitoSOX Red. Results are representative of three independent experiments.

Iron homeostasis and intracellular amount of LIP are both critical for ferroptosis (27, 28). Indeed, high LIP content promotes Fenton reactions thus causing ROS production and ferroptosis; on the other hand, the use of iron chelators protects cancer cells from ferroptosis by restricting the intracellular free iron levels (29–33). We decided to evaluate the LIP content of erastin-sensitive HEY cells and erastin-resistant COV318 cells upon the administration of 8 μM erastin for 8 h. Treatment with 25 μM erastin was excluded due to the excessive cytotoxic effects exerted in HEY cells. Flow cytometry analysis with Calcein-AM staining highlighted two major findings: i) HEY cells showed higher LIP (ΔMFI:3046) compared to COV318 cells (ΔMFI:578) already at baseline conditions, ii) treatment with erastin exacerbates this difference by causing a significant increase of LIP in HEY (ΔMFI:5165) but not in COV318 cells (ΔMFI:545) (**Figure 2A** and **Supplementary Figure S2**). Western blotting analyses suggest that in HEY cells this phenomenon might occur *via* ferritinophagy, since erastin treatment caused a consistent reduction of FtH and its cargo NCOA4 (**Figure 2B**). Conversely, in COV318 cells erastin administration left NCOA4 expression unaltered but rather enhanced FtH expression, thus preventing the overload of intracellular free iron (**Figure 2B**). In parallel, HEY showed a higher baseline content of both total ROS and mitochondrial superoxide species (CM-H2DCFDA MFI:70019; MitoSOX Red MFI:673) compared to COV318 cells (CM-H2DCFDA MFI:40150; MitoSOX Red MFI:484) and erastin treatment strengthened this gap (HEY: CM-H2DCFDA MFI:115691; MitoSOX Red MFI:1067) (COV318: CM-H2DCFDA MFI:39288; MitoSOX Red MFI:641) (**Figure 2C** and **Supplementary Figure S2**).

### Erastin Triggers Ferroptosis in HEY Cells and Causes Mitochondrial Dysfunction

**Figure 3:**
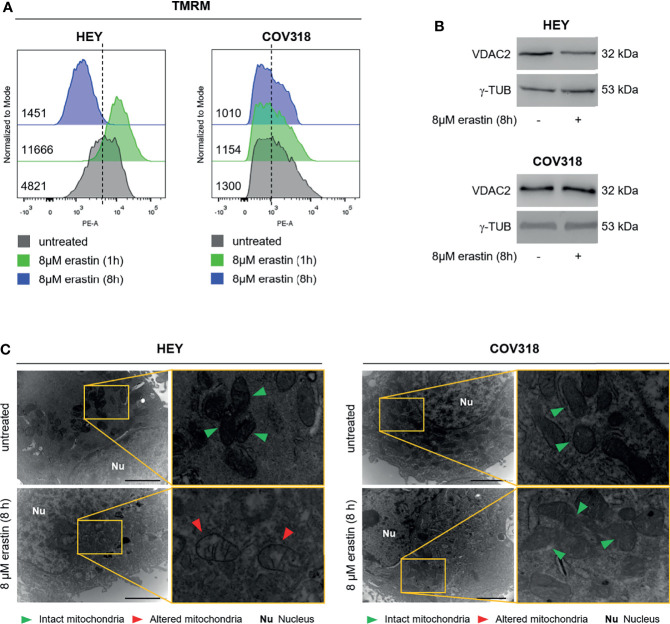
Erastin treatment triggers mitochondrial dysfunction in HEY cells. **(A)** Mitochondrial membrane potential measured by TMRM flow cytometry assay in HEY and COV318 cells untreated or treated with 8 µM erastin for 1 and 8 h. **(B)** Western Blot of VDAC2 in HEY and COV318 cells upon administration of 8 µM erastin for 8 h. γ-TUB was used as a normalization control for protein quantification. **(C)** Mitochondrial ultrastructural images detected by TEM of HEY and COV318 cells treated with or without 8 μM erastin for 8 h. Green arrows, intact mitochondria; red arrows, altered mitochondria; Nu, nucleus. Results are representative of three independent experiments.

Then, we investigated the molecular basis underlying erastin-induced ferroptosis in HEY cells. Erastin treatment may alter mitochondrial membrane potential (ΔΨ), thus ultimately leading to mitochondrial dysfunction (34). Hence, we measured the effects of 8 μM erastin on the ΔΨ by loading HEY cells with the ΔΨ fluorescent indicator TMRM. Flow cytometry analysis revealed that TMRM fluorescence increased in HEY cells within 1h after erastin treatment (MFI:11666) compared to untreated cells (MFI:4821), which is indicative of mitochondrial hyperpolarization, and then significantly decreased after 8 h (MFI:1451) suggesting mitochondrial depolarization (**Figure 3A** and **Supplementary Figure S3A**). Moreover, WB analysis showed that erastin promoted a consistent decrease of the voltage-dependent anion channel 2 (VDAC2), already identified as indicator of ferroptosis-induced mitochondrial dysfunction (35) (**Figure 3B**). In parallel, by using TEM, we observed that erastin determined damage of mitochondria architecture characterized by loss, fragmentation, and disorganization of cristae and a sparse (rarefied) or often vacuolated matrix (red arrowheads in **Figure 3C**). Conversely, in the untreated HEY cells, mitochondria appeared well-preserved with an ordered distribution of cristae (green arrowheads in **Figure 3C**). The same panel of analyses was performed in COV318 cells where the erastin treatment is unable to induce mitochondrial dysfunction (**Figures 3A–C**, **Supplementary Figure S3A**). Finally, albeit a slight reduction of intracellular GPX4 protein levels, lipid peroxidation, measured by both C11-BODIPY and Malondialdehyde (MDA) assays, did not significantly increase neither in HEY nor in COV318 cells treated with erastin (**Supplementary Figures S3B–D**). Nevertheless, in order to evaluate the role of ROS in erastin-induced cell death, we performed a co-treatment with erastin (8 μM for 8 h) and the antioxidant compound trolox (200 μM for 8 h). As reported in **Supplementary Figure S3E**, trolox significantly repressed the erastin-dependent accumulation of both total ROS and mitoROS and, in parallel, consistently abolished the erastin-induced cell death (**Supplementary Figure S3F****).**

### Depletion of Intracellular Free Iron Counteracts Erastin-Induced Ferroptosis in HEY Cells

**Figure 4:**
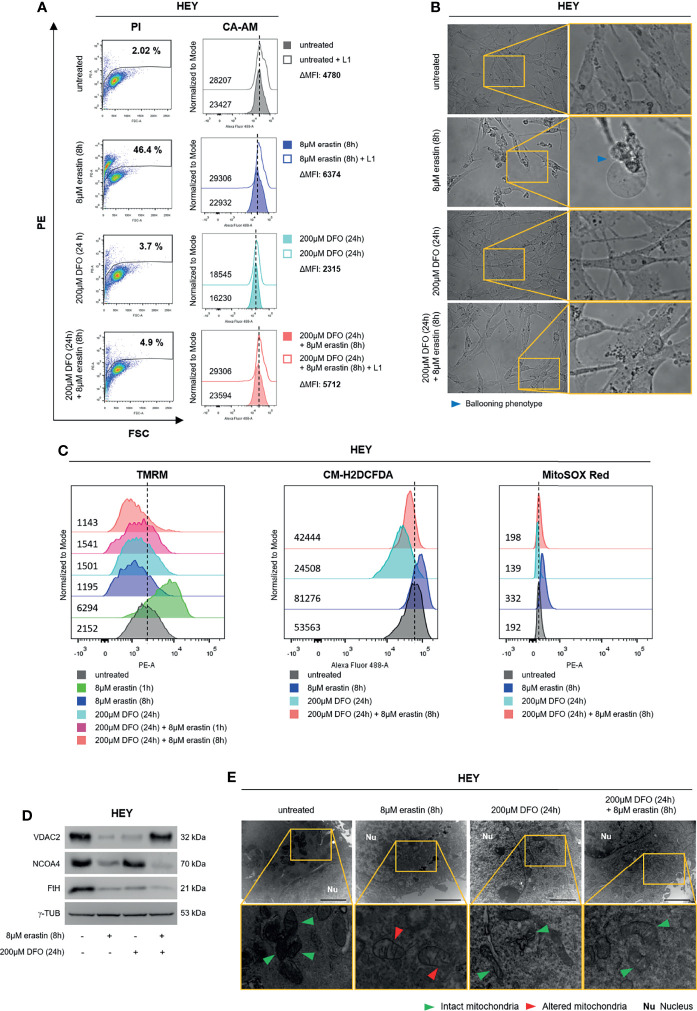
DFO administration protects HEY cells from ferroptosis. **(A)** LIP level and cell mortality determined using CA-AM and PI cytofluorimetric assays in HEY cells untreated, treated with 8 µM erastin (8 h) and 200 µM DFO (24 h) alone or in combination. **(B)** Optical microscopy images showing the presence or absence of the ballooning phenotype in HEY cells upon treatment with erastin and DFO alone or in combination. **(C)** Analysis of mitochondrial membrane potential, cytosolic and mitochondrial ROS production by TMRM, CM-H2DCFDA, and MitoSOX Red fluorescence, respectively, in HEY cells untreated, treated with erastin and DFO alone or in combination. **(D)** Western Blot of VDAC2, NCOA4, and FtH in HEY cells upon administration of erastin and DFO alone or in combination. γ-TUB was used as a normalization control for protein quantification. **(E)** Mitochondrial ultrastructural images detected by TEM in HEY cells upon treatment with erastin and DFO alone or in combination. Green arrows, intact mitochondria; red arrows, altered mitochondria; Nu, nucleus. All data are representative of three independent experiments.

To further deepen the role of baseline LIP amount in erastin-triggered ferroptosis, HEY cells were first treated with the iron chelator deferoxamine mesylate (DFO) (200 μM) for 24 h; then, culture medium was replaced, and cells were treated with erastin (8 μM for 8 h). First, DFO treatment alone significantly reduced the LIP (HEY^DFO^ ΔMFI:2315 vs HEY^untreated^ ΔMFI:4780), as well as both total and mitoROS (HEY^DFO^ CM-H2DCFDA:24508 vs HEY^untreated^ CM-H2DCFDA:53563; HEY^DFO^ MitoSOX Red:139 vs HEY^untreated^ MitoSOX Red:192). Albeit leading to a slight mitochondrial membrane depolarization (HEY^DFO^ TMRM MFI:1501 vs HEY^untreated^ TMRM MFI:2152) and reduction of VDAC2 protein levels, DFO alone did not affect mitochondrial ultrastructure or HEY cell death (3.7% PI^+^ HEY^DFO^ vs 2.02% PI^+^ HEY^untreated^) (**Figures 4A–E**). Then, we observed that the reduction of intracellular free iron strongly protected HEY cells from erastin-induced cell death (HEY^untreated^ ΔMFI:4780, HEY^erastin^ ΔMFI:6374, HEY^DFO/erastin^ ΔMFI:5712) (2.02% PI^+^ HEY^untreated^, 46.4% PI^+^ HEY^erastin^, 4.9% PI^+^ HEY^DFO/erastin^) (**Figures 4A** and **Supplementary Figure S4**). Accordingly, HEY cells pre-treated with DFO did not show the “ballooning” phenotype upon administration of erastin (**Figure 4B**), and mitochondrial functionality appeared overall preserved. Indeed, i) total and mitoROS were inhibited by the iron chelation (HEY^untreated^ CM-H2DCFDA:53563, HEY^erastin^ CM-H2DCFDA:81276, HEY^DFO/erastin^ CM-H2DCFDA:42444) (HEY^untreated^ MitoSOX Red:192, HEY^erastin^ MitoSOX Red:332, HEY^DFO/erastin^ MitoSOX Red:198) (**Figures 4C** and **Supplementary Figure S4**); ii) DFO significantly counteracted the mitochondrial membrane hyperpolarization induced by 1 h erastin and then left the ΔΨ almost unaltered upon 8 h erastin (HEY^untreated^ TMRM MFI:2152; HEY^erastin (1 h)^ TMRM MFI:6294; HEY^erastin (8 h)^ TMRM MFI:1195; HEY^DFO/erastin (1 h)^ TMRM MFI:1541; HEY^DFO/erastin (8 h)^ TMRM MFI:1143) (**Figures 4C** and **Supplementary Figure S4**); iii) erastin-mediated VDAC2 downregulation was consistently counteracted by DFO while FtH and NCOA4 levels continued to decrease (**Figure 4D**); iv) mitochondrial ultrastructure was maintained (**Figure 4E**).

Overall, these results indicate that ferroptosis sensitivity of HEY cells is associated with the baseline LIP and that a sufficient increase of intracellular free iron upon erastin treatment is mandatory to trigger mitochondrial dysfunction.

### Ferlixit Sensitizes COV318 Cells to Erastin-Mediated Ferroptosis

**Figure 5:**
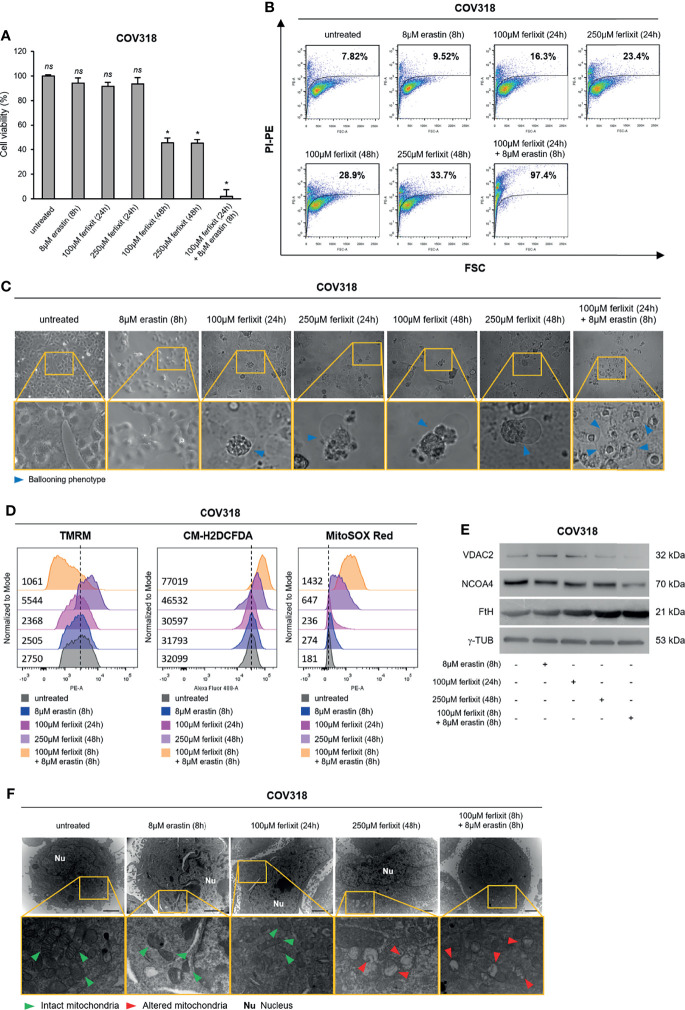
Ferlixit/erastin co-treatment leads to ferroptosis in COV318 cells. COV318 cells were treated with different concentrations (100 and 250 µM for 24 and 48 h) of ferlixit alone or in combination with 8 µM erastin for 8 h. Cell viability and mortality were measured using the MTT assay **(A)** and the PI flow cytometry assay **(B)**, respectively. Results are shown as means ± SD of three independent experiments. *p-value <0.05, untreated vs treatment; ns, not significant. **(C)** Optical microscopy images showing the presence or absence of the ballooning phenotype in COV318 cells upon treatment with ferlixit and erastin alone or in combination. **(D)** Analysis of mitochondrial membrane potential, cytosolic and mitochondrial ROS production by TMRM, CM-H2DCFDA and MitoSOX Red flow cytometry assays in COV318 cells untreated, treated with ferlixit and erastin alone or in combination. **(E)** Western Blot of VDAC2, NCOA4, and FtH in COV318 cells upon administration of ferlixit and erastin alone or in combination. γ-TUB was used as a normalization control for protein quantification. **(F)** Mitochondrial ultrastructural images detected by TEM in COV318 cells upon treatment with ferlixit and ferlixit alone or in combination. Green arrows, intact mitochondria; red arrows, altered mitochondria; Nu, nucleus. All data are representative of three independent experiments.

Then, to demonstrate that a sufficient baseline LIP is essential to induce ferroptosis, we treated the erastin-resistant COV318 cells with growing concentrations of ferlixit (100 and 250 μM for 24 and 48 h). Plus, the lowest dose of ferlixit (100 μM) was used as pre-treatement for 24 h followed by administration of 8 μM erastin (8 h). We observed that the sole administration of ferlixit at 250 μM for 48 h reduced COV318 cell viability to ~45% (*p <*0.05) and that the combination (100 μM for 24 h) with erastin (8 μM for 8 h) broke down cell viability to less than 2% (*p <*0.05) (**Figure 5A**). In agreement, cytofluorimetric assays show that PI^+^ COV318 cells upon 250 μM for 48 h were 33.7% while PI^+^ COV318 cells treated with the combined therapy reached 97.4% (**Figure 5B**). Consistently with these effects, the ballooning phenotype increased along with the growing concentrations and time of ferlixit treatment, and then reaches its maximum upon the combination of ferlixit and erastin (**Figure 5C**). Since the synergy of ferlixit (100 μM, 24 h) with erastin (8 μM, 8 h) led to excessive cytotoxic effects, the analyses of the molecular mechanisms underlying the combination therapy approach were performed shortening the time of ferlixit treatment (100 μM, 8 h). First, we demonstrated that ferlixit/erastin combined treatment caused a greater increase of LIP compared to ferlixit alone (250 μM, 48 h) (COV318^untreated^ ΔMFI:9315, COV318^250 µMferlixit^ ΔMFI:18616, COV318^ferlixit/erastin^ ΔMFI:45345) (**Supplementary Figure S5A**). In parallel, total and mitochondrial ROS already rise upon ferlixit alone and underwent a further increase after the combined therapy (COV318^untreated^ CM-H2DCFDA:32099, COV318^erastin^ CM-H2DCFDA:31793, COV318^100 µMferlixit^ CM-H2DCFDA:30597, COV318^250 µMferlixit^ CM-H2DCFDA:46532, COV318^ferlixit/erastin^ CM-H2DCFDA:77019) (COV318^untreated^ MitoSOX Red:181, COV318^erastin^ MitoSOX Red:274, COV318^100 µMferlixit^ MitoSOX Red:236, COV318^250 µMferlixit^ MitoSOX Red:647, COV318^ferlixit/erastin^ MitoSOX Red:1432) (**Figures 5D** and **Supplementary Figure S5B**). Notably, when ferlixit was used alone at 250 μM for 48 h, the ΔΨ significantly increased while when used in combination with erastin mitochondria faced membrane depolarization (COV318^untreated^ TMRM:2750, COV318^erastin^ TMRM:2505, COV318^100 µMferlixit^ TMRM:2368, COV318^250 µMferlixit^ TMRM:5544, COV318^ferlixit/erastin^ TMRM:1061) (**Figures 5D** and **Supplementary Figure S5B**). The WB analysis revealed that while the sole ferlixit administration (250 μM, 48 h) caused a slight decrease of VDAC2, this reduction became consistent only upon the combined treatment. The effects of both therapeutic approaches on FtH and NCOA4 deserve more detailed comments: i) FtH resulted always upregulated because ferlixit, as any other iron compound, promotes its translation through the IRP/IRE iron regulatory system; ii) NCOA4 is unaffected by ferlixit alone, but it appears downregulated upon ferlixit/erastin treatment (**Figure 5E**). At last, TEM analysis highlights that ferlixit promoted an initial mitochondrial morphology alteration while the combined treatment provoked evident mitochondria disorganization (**Figure 5F**). Once again, no increase in lipid peroxidation has been detected (**Supplementary Figures S5C–E**).

### Ferlixit Alone Mimics Erastin Effects and Promotes Ferroptosis in HEY Cells

**Figure 6:**
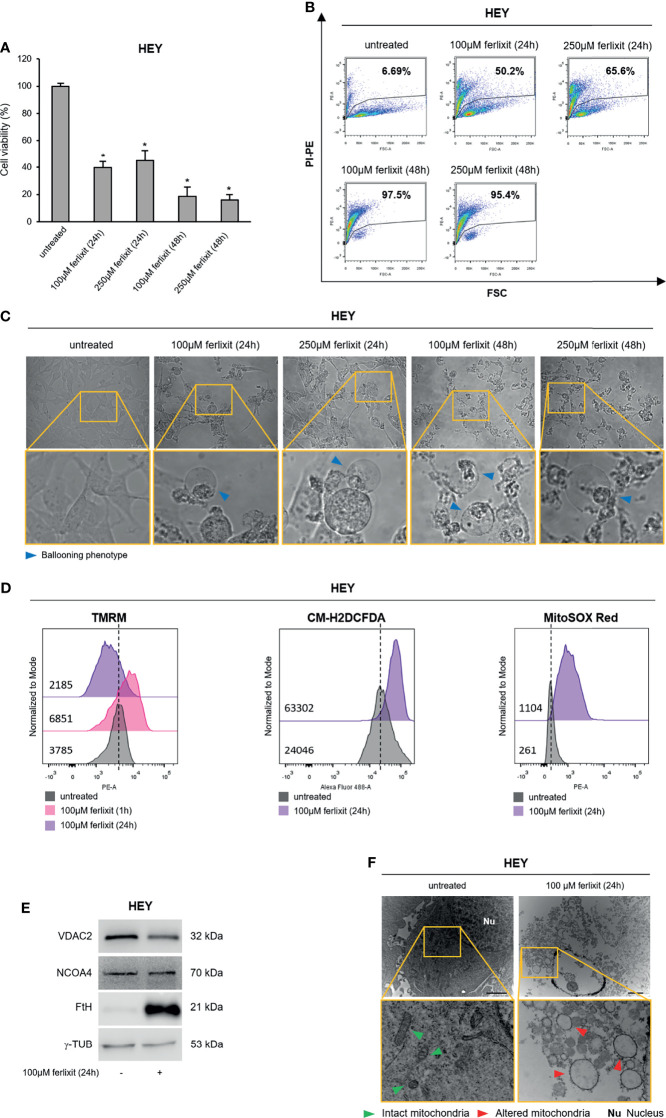
Ferlixit admnistration alone induces ferroptosis in HEY cells. HEY cells were treated with different concentrations (100 and 250 µM) of ferlixit for 24 and 48 h. Cell viability and mortality were measured using the MTT assay **(A)** and PI flow cytometry assay **(B)**, respectively. Results are shown as means ± SD of three independent experiments. *p-value <0.05, untreated vs treatment. **(C)** Optical microscopy images showing the presence or absence of the ballooning phenotype in HEY cells upon treatment with different concentration of ferlixit. **(D)** Analysis of mitochondrial membrane potential, cytosolic and mitochondrial ROS production by TMRM, CM-H2DCFDA and MitoSOX Red flow cytometry assays in HEY cells untreated or treated with 100 µM ferlixit for 24 h. **(E)** Western Blot of VDAC2, NCOA4 and FtH in HEY cells upon administration of 100 µM ferlixit for 24 h. γ-TUB was used as a normalization control for protein quantification. **(F)** Mitochondrial ultrastructural images detected by TEM in HEY cells treated with 100 µM ferlixit for 24 h. Green arrows, intact mitochondria; red arrows, altered mitochondria; Nu, nucleus. All data are representative of three independent experiments.

Finally, we wondered whether in the ferroptosis-sensitive HEY cells, characterized by a consistent baseline LIP, the sole administration of ferlixit might totally replace the use of erastin. Thus, we treated HEY cells with ferlixit (100 and 250 μM for 24 and 48 h) and we found that already at the lowest concentration cell viability decreased to ~40% (*p <*0.05) and the relative PI^+^ HEY cells were 50.2%. Notably, at 100 and 250 μM for 48 h, ferlixit caused massive cytotoxic effects (PI^+^ HEY^100 μM ferlixit (48 h)^: 97.5%; PI^+^ HEY^250 μM ferlixit (48 h)^: 95.4%) (**Figures 6A, B**). The ballooning phenotype clearly mirrored the above-mentioned effects (**Figure 6C**). Once we demonstrated the increase in the amount of intracellular LIP (**Supplementary Figure S6A**), we selected the lowest dose of ferlixit (100 μM for 24 h) to explore the underlying molecular mechanisms. Flow cytometry analysis revealed that TMRM fluorescence increased in HEY cells within 1 h after ferlixit treatment (MFI:6851) and then decreased after 24 h (MFI:2185) compared to untreated cells (MFI:3785), which is indicative of the two main step of mitochondrial hyperpolarization and depolarization. In agreement, i) total and mitochondrial ROS increased upon ferlixit administration (HEY^untreated^ CM-H2DCFDA:24046 vs HEY^ferlixit^ CM-H2DCFDA:63302) (HEY^untreated^ MitoSOX Red:261 vs HEY^ferlixit^ MitoSOX Red:1104) (**Figures 6D** and **Supplementary Figure S6B**), ii) VDAC2 was downregulated (**Figure 6E**). Here too, ferlixit induced FtH upregulation while NCOA4 resulted unaltered (**Figure 6E**). Mitochondria morphological analysis by TEM highlights that in this cell type the sole ferlixit was able to promote mitochondria swelling (**Figure 6F**). Once again, no increase in lipid peroxidation has been observed (**Supplementary Figures S6C–E**).

In order to stress the potential use of iron compounds as single agents or combination therapy with erastin to sensitizes OVCA cells to ferroptosis, we treated both HEY and COV318 cells with 10 μM hemin. As shown in **Supplementary Figure S6F**, 10 μM hemin for 24 h was sufficient to trigger ferroptosis in HEY cells (78.1% PI^+^ HEY) while treatment with 10 μM for 48 h moderately promoted cell death in COV318 cells (34.6% PI^+^ COV318). Therefore, we combined hemin (10 μM for 48 h) with erastin (8 μM for 8 h) and we observed a significant impact on cell death also in COV318 (94.7% PI^+^ COV318). The analysis of the “ballooning phenotype” further supported the role of hemin in ferroptotic cell death (**Supplementary Figure S6G**).

## Discussion

During the last decade, novel molecular-targeted agents along with combination therapies have been developed and approved for the management of ovarian tumors; however, the prognosis of females suffering OVCA still remains unsatisfactory since the majority of them develop chemoresistance (36). Among the various mechanisms underlying acquired drug resistance, reduced susceptibility to apoptosis is one of the most important (37), therefore representing the main actual challenge of OVCA research. In this scenario, identification of agents able to induce ferroptosis is in the spotlight.

Ferroptosis is a programmed cell death that significantly differs from apoptosis in terms of both morphology and biochemical pathways (38). The main feature of ferroptosis is the accumulation of intracellular free iron causing membrane lipid peroxidation, increased intracellular ROS and mitochondrial dysfunction (27). Erastin, the first FIN identified to selectively induce a caspase-independent cell death, acts on all the above mentioned mechanisms (39). It may inhibit the cysteine/glutamate antiporter (system Xc-), thus blocking cysteine uptake and glutathione (GSH) synthesis. This phenomenon indirectly breaks down the activity of the antioxidant enzyme GPX4 and leads to the accumulation of peroxides and hydroxyl radicals and to the peroxidation of polyunsaturated fatty acids (PUFAs) in cell membrane (39). Besides, erastin may target VDACs by inducing their degradation (35) or by antagonizing the effects of free tubulin and thus promoting their opening (40). In both cases, erastin may promotes the increase of mitochondrial membrane potential and accumulation of mitoROS (41, 42).

To enable growth, certain cancer cells exhibit a more pronounced iron demand (43). The downside is that the iron dependency at the same time makes cancer cells more vulnerable to ferroptosis, which therefore might be considered as a druggable “Achilles heel”. Several ferroptosis inducers have been so far identified and approved with the expectation to bypass the disadvantages of traditional chemotherapies based on induction of apoptosis (44, 45). Numerous studies have shown that ferroptosis has consistent antitumor effects or, alternatively, enhances chemosensitivity in OVCA (21). However, a deep investigation of this field has revealed that i) different ovarian tumor cells exhibit different degree of susceptibility and ii) multiple mechanisms of resistance and a poor pharmacokinetics may limit the effectiveness of FINs *in vivo.* Therefore, the use of ferroptosis inducers as novel therapeutic strategies in OVCA must definitely take these two aspects into consideration.

In this study, we demonstrate that erastin fails to trigger ferroptosis in diverse OVCA cell lines and that this essentially occurs when both the baseline and the treatment-induced intracellular levels of LIP are inadequate to cause a detrimental oxidative stress. Furthermore, we show that the combination of erastin with the iron compound ferlixit might overcome this vulnerability and improve treatment results in OVCA.

First, we found that among the OVCA cell lines included in the study, erastin exerts cytotoxic effects in HEY and A2780 but not in COV318 and PEO4 cells, thus indicating that erastin-susceptibility is independent, in these cells, from either *KRAS* mutational status or sensitivity to platinum-based chemotherapy. Rather, we observed that erastin-sensitive HEY cells are characterized by higher LIP and ROS content compared to the erastin-resistant COV318 cells already at baseline conditions. This data confirms what previously suggested by Torti’s group, which is that “iron-addiction” phenotype, characterized by high constitutive intracellular LIP, boosts the susceptibility of OVCA cells to ferroptosis (15). Furthermore, we found that HEY cells show a significantly higher growth rate compared to COV318 (**Supplementary Figure S7**). Based on our data, we cannot say whether the “iron addiction” is directly or indirectly linked to the higher cell growth potential of HEY cells. However, our results are in agreement with a preliminary study demonstrating that erastin and RSL3 preferentially trigger ferroptosis in highly proliferating rhabdomyosarcoma and myoblast cells (46). This aspect deserves additional works.

A second aspect that comes out from our data is that erastin might trigger different pathways in different cell types. Erastin, exclusively in HEY cells, aggravates the intracellular oxidative stress by inducing the NCOA4-mediated degradation of ferritin. Ferritin, with its iron-storage function, is one of the major proteins involved in the maintenance of intracellular iron homeostasis and in the protection against ROS generation (23, 24, 47–52). Under certain conditions, the protein cargo NCOA4 binds and delivers ferritin to the autophagosome where, after lysosomal degradation, it releases iron into the cytosol thus increasing LIP and ROS accumulation (53). NCOA4-mediated ferritinophagy may contribute to erastin-induced ferroptosis, as recently reported in human fibrosarcoma and pancreatic cancer (53, 54). This pattern appears to be active also in HEY cells, where we found that the treatment leads to NCOA4 and FtH proteins downregulation accompanied by a surge in both intracellular free iron and ROS content. Indeed, either Fer-1 or DFO consistently inhibited the erastin-induced cell death. In stark contrast, in the resistant COV318 cells erastin treatment not only leaves NCOA4 amounts unaltered but also promotes a consistent upregulation of FtH protein levels, which consequently mitigate further increase of LIP and ROS. The different behavior of HEY and COV318 cells might be attributed to several possible causes. One can be found in the molecular mechanisms regulating intracellular iron homeostasis: in COV318 cells the increase of intracellular free iron, secondary to ferritinophagy, may engage the IRE/IRP machinery leading to the upregulation of ferritin synthesis as a mechanism of cell self-protection (55). Alternatively, erastin may promote FtH transcription through the activation of the transcriptional factor Nrf-2 or through the downregulation of YAP pathway as very recently demonstrated in hepatocellular carcinoma and lung adenocarcinoma, respectively (13, 56). Which of the aforementioned or some yet-unknown mechanisms are involved in the restriction of ferroptosis in COV318 will be deepened in a future work.

Notably, we demonstrate that the use of ferlixit, an iron compound (Fe^3+^) normally used to treat anemia, significantly sensitizes OVCA cells to ferroptosis either alone or in combination with erastin. Ferlixit-induced ferroptotic cell death was confirmed by the use of the specific ferroptosis inhibitor Fer-1 (**Supplementary Figure S8**). Moreover, we feel confident to exclude the occurrence of either apoptosis or autophagy. Indeed, i) ferlixit alone or in combination with erastin did not cause alteration of *p53* and *BAX* expression levels and ii) the use of autophagy inhibitor (Cq) did not interfere with the cytotoxic effect of the two drugs alone or used as combined treatment (**Supplementary Figure S8**). It is worthy to note that in HEY cells, the sole ferlixit administration distinctly causes ferroptosis. In COV318 cells, instead, it is necessary to combine ferlixit and erastin to readily induce a detrimental effect in more than 95% of cells. We hypothesize that in HEY cells, ferlixit alone mimics the increase of LIP in an autophagy-independent manner. In COV318 cells, it is most likely that the combination of ferlixit and erastin leads to an increase of LIP to an extent that evades the still not decoded protective feedback mechanisms. Ferroptosis is overall associated with multiple molecular pathways (4, 39). In the current study, we demonstrate that erastin and ferlixit alone or used as combined therapy downregulate VDAC2 on the outer mitochondrial membrane thus impairing the ΔΨ and damaging mitochondrial morphology. Conversely, although lipid peroxidation is considered the main hallmark of ferroptosis (57), no increase of intracellular lipid hydroperoxides has been observed. For the moment, we do not have a clear explanation about the molecular basis underlying this “non-canonical” pathway; however, the two recent studies also highlighted that erastin treatment may promote ferroptosis in gastric and lung cancer without impairing the lipid peroxidation homeostasis (58, 59). Future focused studies are necessary to provide mechanistic insights into this odd.

Overall, our findings show that both the baseline and the treatment-induced LIP content determines the response of OVCA cells to erastin-mediated ferroptosis. In particular, ferroptosis occurs when LIP reaches a threshold level causing a detrimental oxidative stress. Furthermore, we demonstrate the administration of low doses of ferlixit improves the effect of erastin-based antitumor approach and, thus, put the ground for the use of this combined therapy in future *in vivo* studies. Taking into account that, so far, the use of FINs *in vivo* has been limited by unsatisfactory pharmacokinetic and physicochemical properties (20), the identification of a reasonable combination strategy involving ferroptosis inducers and other compounds may enhance treatment efficacy, reduce the therapeutic dose of each individual drug and minimize the adverse reactions at once.

## Data Availability Statement

The original contributions presented in the study are included in the article/**Supplementary Material**. Further inquiries can be directed to the corresponding author.

## Author Contributions

FC and FB designed this study. AMB, AS, and MS performed the analysis. IDP performed electron microscopy analysis. FC and FB wrote the manuscript. AMB, MCF, DT, SL, FC, and FB reviewed the manuscript. All authors listed have made a substantial, direct, and intellectual contribution to the work and approved it for publication.

## Conflict of Interest

The authors declare that the research was conducted in the absence of any commercial or financial relationships that could be construed as a potential conflict of interest.

## Publisher’s Note

All claims expressed in this article are solely those of the authors and do not necessarily represent those of their affiliated organizations, or those of the publisher, the editors and the reviewers. Any product that may be evaluated in this article, or claim that may be made by its manufacturer, is not guaranteed or endorsed by the publisher.
