# Supplementary material for: Iron Administration Overcomes Resistance to Erastin-Mediated Ferroptosis in Ovarian Cancer Cells
Source: Front Oncol. 2022 Mar 31;12:868351. doi: 10.3389/fonc.2022.868351 (PMC9008715; doi:10.3389/fonc.2022.868351)
Supplement: Supplementary file 1 [file DataSheet_1.pdf]

Supplementary Material

Iron administration overcomes resistance to erastin-mediated ferroptosis in ovarian cancer cells

Battaglia AM et al.

Supplementary Figure 1

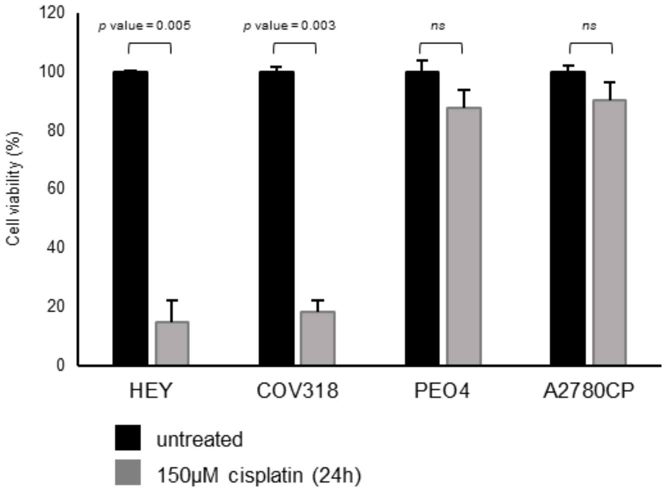

Supplementary Figure 1. Cell viability analysis of HEY, COV318, PEO4 and A2780CP OVCA cells upon treatment with 150µM cisplatin for 24h. Results are shown as means ± SD of three independent experiments. *p* value < 0.05 was considered as statistically significant. ns: not significant.

Supplementary Figure 2

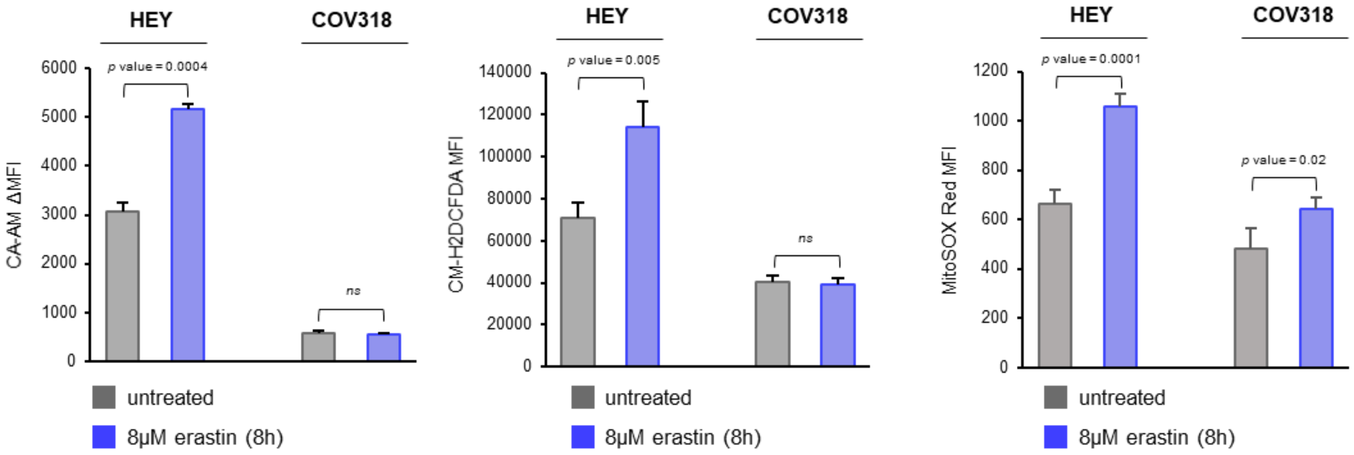

Supplementary Figure 2. Relative histograms of flow cytometry analysis of LIP level, cytosolic and mitochondrial ROS production by using CA-AM, CM-H2DCFDA and MitoSOX Red assays in HEY and COV318 cells untreated and treated with 8µM erastin for 8h. Results are shown as means ± SD of three independent experiments. p value < 0.05 was considered as statistically significant. ns: not significant.

Supplementary Figure 3

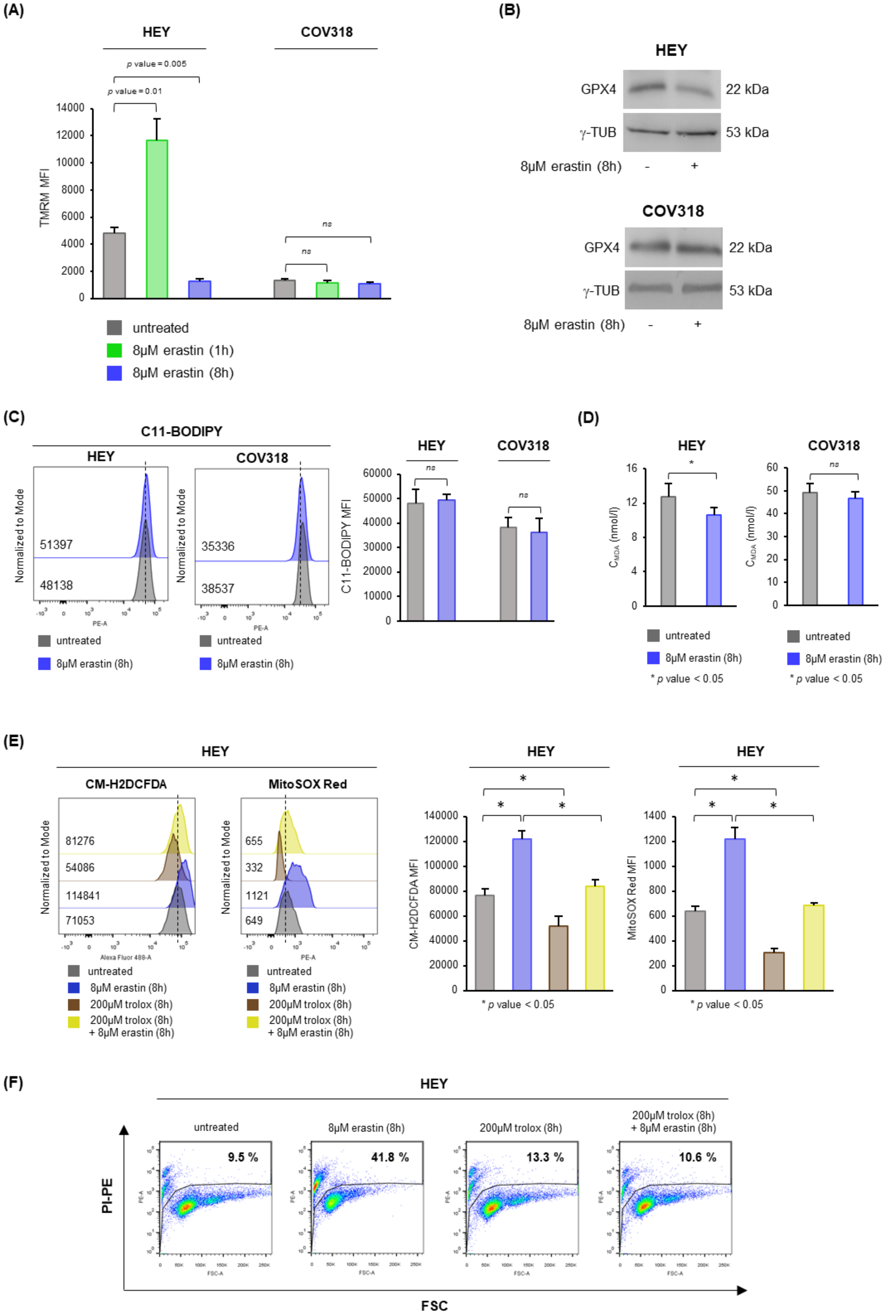

Supplementary Figure 3. (A) Relative histograms of flow cytometry analysis of mitochondrial membrane potential by using TMRM assay in HEY and COV318 cells untreated and treated with 8µM erastin for 1 and 8h. **(B)** Western Blot of GPX4 in HEY and COV318 cells upon administration of 8µM erastin for 8h. γ-TUB was used as a normalization control for protein quantification. Analysis of lipid peroxidation by both flow cytometry using C11-BODIPY **(C)** and MDA assay **(D)** in HEY and COV318 cells untreated or treated with 8µM erastin for 8h. **(E)** Analysis of cytosolic and mitochondrial ROS production by CM-H2DCFDA and MitoSOX Red fluorescence assays and relative histograms, in HEY cells untreated, treated with 8µM erastin (8h) and 200µM trolox (8h) alone, and in combination. **(F)** Cell mortality determined using PI cytofluorimetric assay in HEY cells untreated, treated with erastin and trolox alone, and in combination. All data are presented as mean ± SD from three independent experiments. p value < 0.05 was considered as statistically significant. ns: not significant.

Supplementary Figure 4

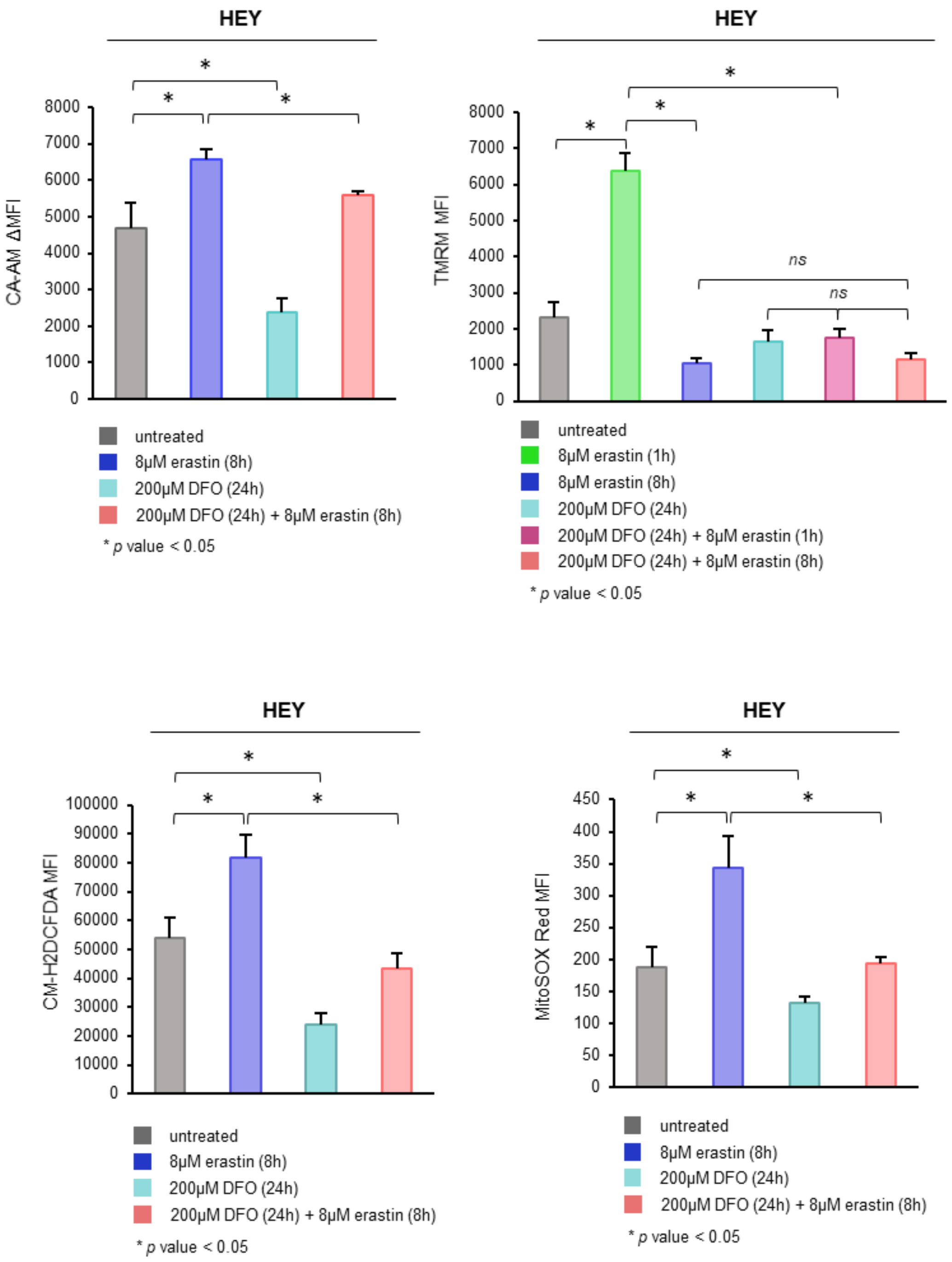

Supplementary Figure 4. Relative histograms of flow cytometry analysis of LIP level, mitochondrial membrane potential, cytosolic and mitochondrial ROS production by using CA-AM, TMRM, CM-H2DCFDA and MitoSOX Red assays in HEY cells untreated, treated with 8µM erastin (8h) and 200µM DFO (24h) alone, and in combination. Results are shown as means ± SD of three independent experiments. p value < 0.05 was considered as statistically significant. ns: not significant.

Supplementary Figure 5

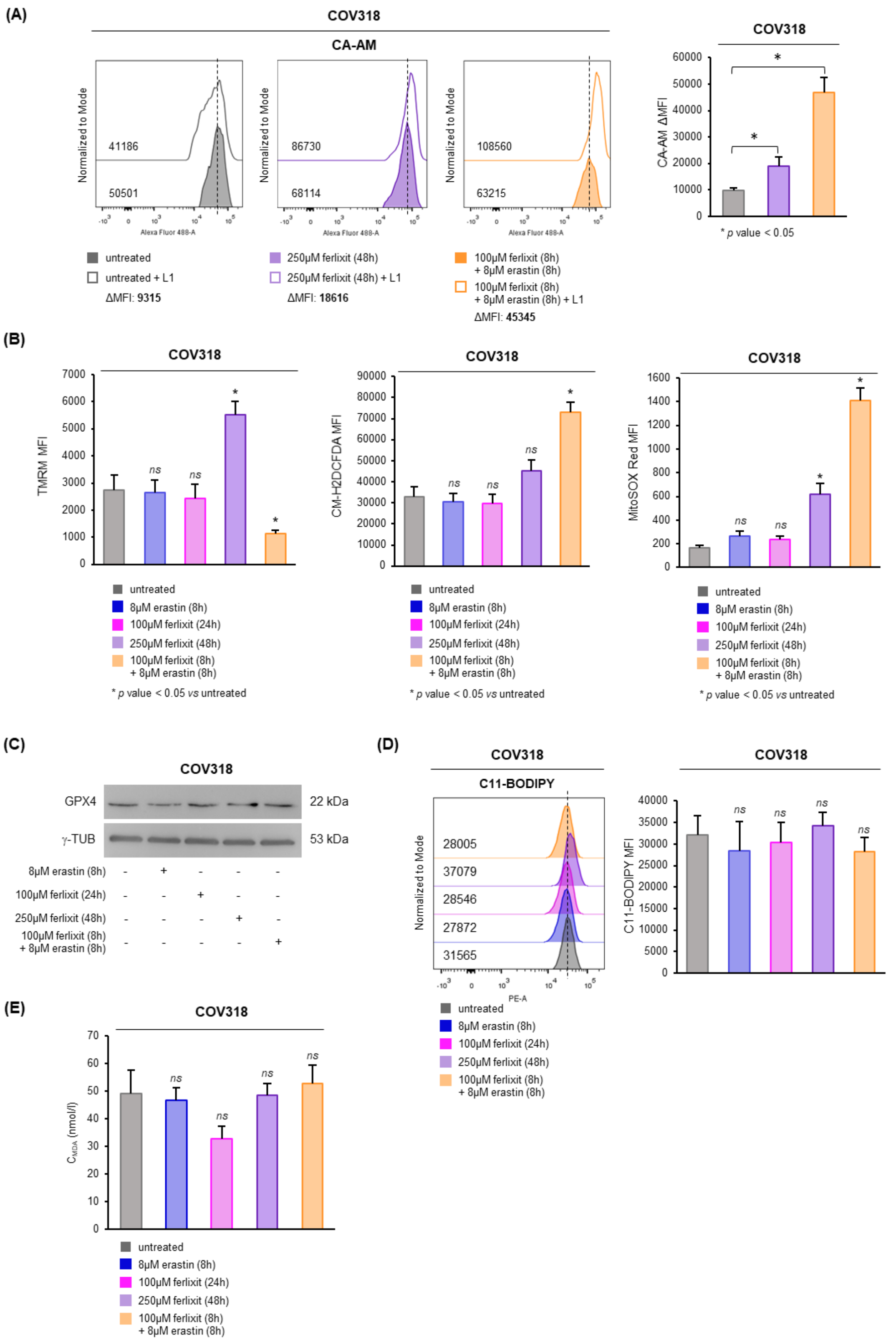

Supplementary Figure 5. (A) LIP level assessed by flow cytometry using CA-AM and relative histogram, in COV318 cells upon treatment with ferlixit and erastin alone, and in combination. **(B)** Relative histograms of flow cytometry analysis of mitochondrial membrane potential, cytosolic and mitochondrial ROS production by using TMRM, CM-H2DCFDA and MitoSOX Red assays in COV318 cells untreated and treated with ferlixit and erastin alone, and in combination. **(C)** Western Blot of GPX4 in COV318 cells upon administration of ferlixit and erastin alone, and in combination. γ-TUB was used as a normalization control for protein quantification. Analysis of lipid peroxidation by both flow cytometry using C11-BODIPY **(D)** and MDA assay **(E)** in COV318 cells untreated, treated with ferlixit and erastin alone, and in combination. All data are presented as mean ± SD from three independent experiments. p value < 0.05 was considered as statistically significant. ns: not significant.

Supplementary Figure 6

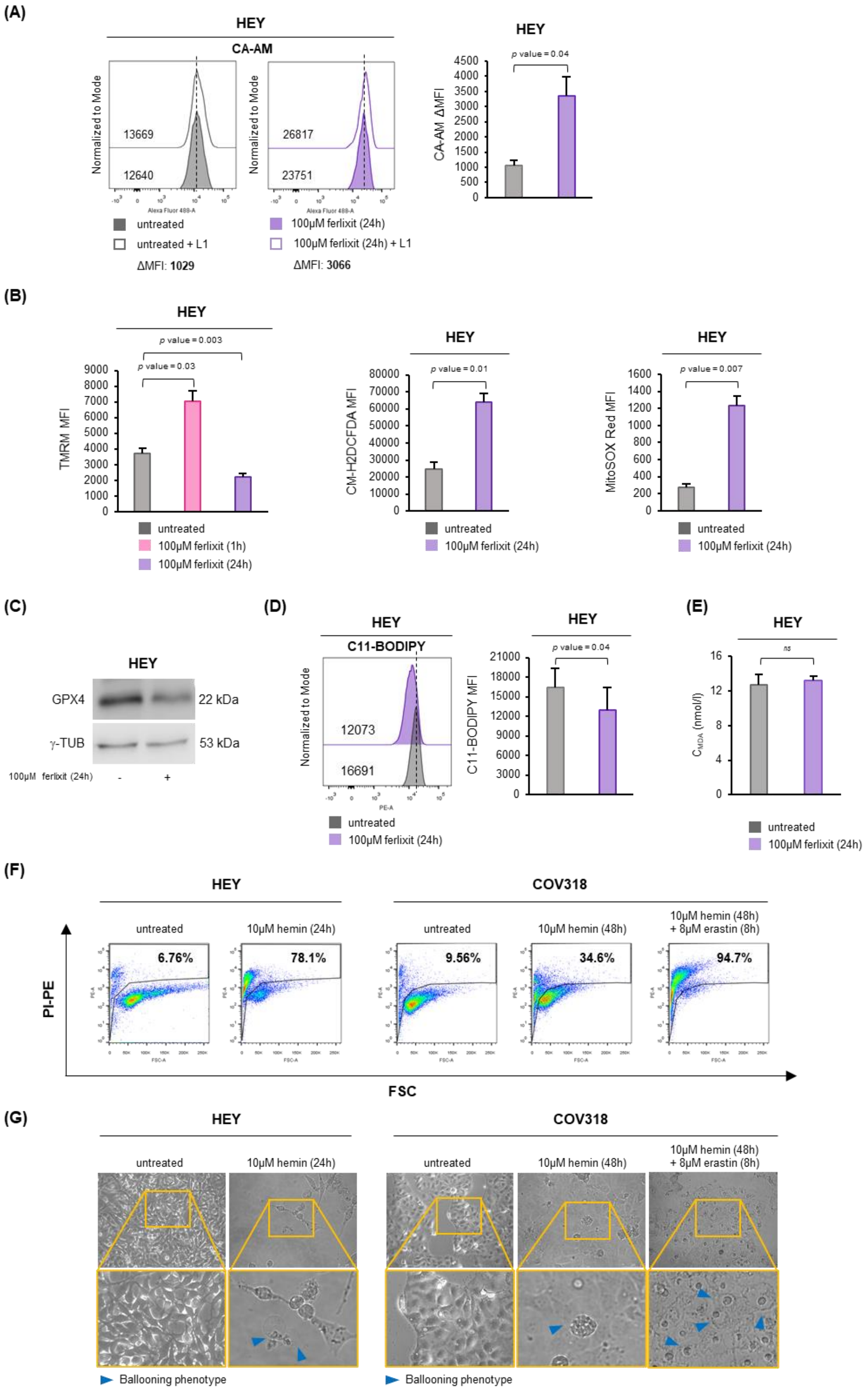

Supplementary Figure 6. **(A)** LIP level assessed by flow cytometry using CA-AM and relative histogram, in HEY cells upon administration of 100µM ferlixit for 24h. **(B)** Relative histograms of flow cytometry analysis of mitochondrial membrane potential, cytosolic and mitochondrial ROS production by using TMRM, CM-H2DCFDA and MitoSOX Red assays in HEY cells untreated and treated with ferlixit. **(C)** Western Blot of GPX4 in HEY cells upon administration of 100µM ferlixit for 24h. γ-TUB was used as a normalization control for protein quantification. Analysis of lipid peroxidation by both flow cytometry using C11-BODIPY **(D)** and MDA assay **(E)** in HEY cells untreated or treated with 100µM ferlixit for 24h. **(F)** Cell mortality determined using PI cytofluorimetric assay in HEY and COV318 cells untreated, treated with 10µM hemin alone and/or co-treated with ferlixit. **(H)** Optical microscopy images showing the presence or absence of the ballooning phenotype in HEY and COV318 cells untreated, treated with 10µM hemin alone and/or co-treated with ferlixit. All data are presented as mean ± SD from three independent experiments. *p* value < 0.05 was considered as statistically significant. ns: not significant.

Supplementary Figure 7

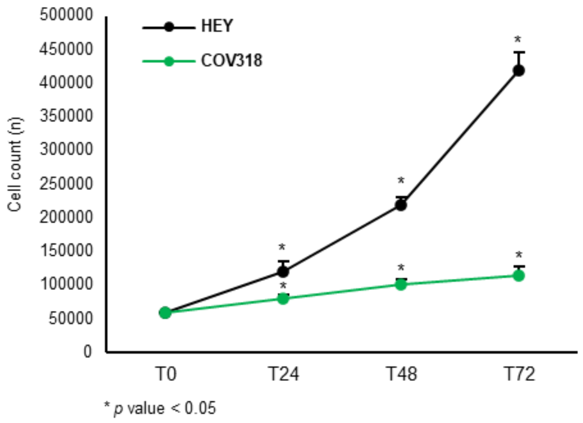

Supplementary Figure 7. Growth rate analysis of HEY and COV318 cells by using Trypan Blue assay. Results are shown as means ± SD of three independent experiments. *p* value < 0.05 was considered as statistically significant. ns: not significant.

Supplementary Figure 8

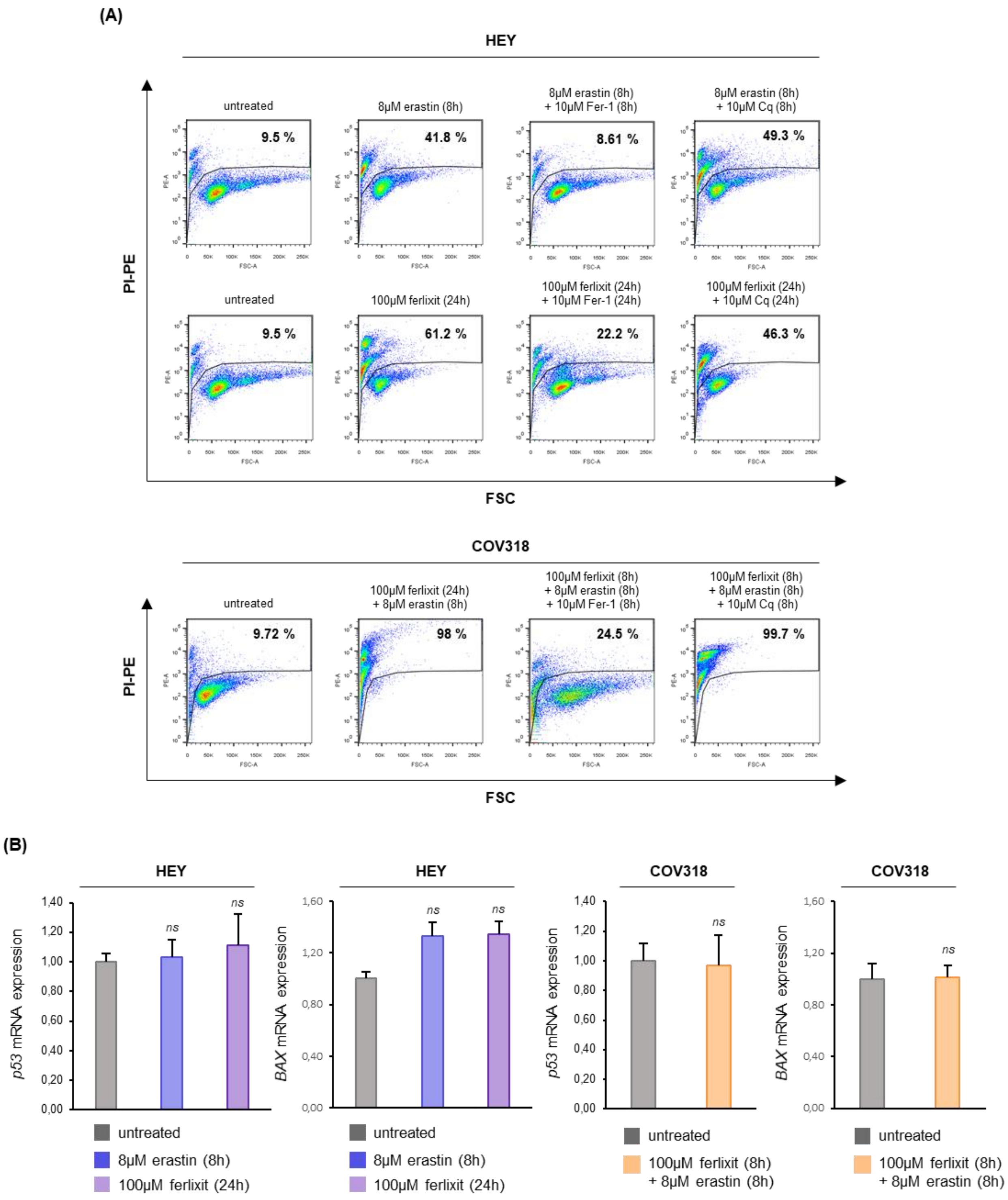

Supplementary Figure 8. (A) Cell mortality determined using PI cytofluorimetric assay in HEY and COV318 cells untreated, treated with erastin and/or ferlixit and co-treated with 10µM Fer-1 and/or 10µM Cq. **(B)** *p53* and *BAX* mRNA levels in HEY and COV318 cells untreated and treated with erastin and/or ferlixit. All data are presented as mean ± SD from three independent experiments. p value < 0.05 was considered as statistically significant. ns: not significant.
